# Oxymatrine Attenuates Ulcerative Colitis through Inhibiting Pyroptosis Mediated by the NLRP3 Inflammasome

**DOI:** 10.3390/molecules29122897

**Published:** 2024-06-18

**Authors:** Jing Sun, Shuai Wang, Zhengtian Zhao, Jiaqi Lu, Yiming Zhang, Wen An, Wei Li, Li Yang, Xiaowei Tong

**Affiliations:** 1 State Key Laboratory of Fine Chemicals, Department of Pharmaceutical Engineering, School of Chemical Engineering, Dalian University of Technology, No. 2, Linggong Road, Ganjingzi District, Dalian 116024, China13072454145@163.com (X.T.); 2 Ningbo Institute of Dalian University of Technology, No. 26, Yucai Road, Jiangbei District, Ningbo 315016, China

**Keywords:** oxymatrine, ulcerative colitis, NLRP3 inflammasome, pyroptosis, GSDMD

## Abstract

Ulcerative colitis (UC) is difficult to cure and easy to relapse, leading to poor quality of life for patients. Oxymatrine (OMT) is one of the main alkaloids of *Sophora flavescens* Aiton, which has many effects, such as anti-inflammation, anti-oxidative stress, and immunosuppression. This study aimed to investigate whether OMT could attenuate ulcerative colitis by inhibiting the NOD-like receptor family pyrin domain containing three (NLRP3) inflammasome-mediated pyroptosis. In this study, the UC rat models were established by 2,4,6-Trinitrobenzenesulfonic acid (TNBS) in vivo, while RAW264.7 cells and peritoneal macrophages were stimulated with Lipopolysaccharides/Adenosine Triphosphate (LPS/ATP) in vitro to simulate pyroptosis models, and Western blotting (WB) and other detection techniques were applied to analyze proteins involved in the NLRP3 inflammasome pathway. Our results showed that OMT alleviated colitis ulcers and pathological damage in the TNBS-induced UC rats and exhibited an inhibitory effect on pyroptosis at the early stage of UC. In the model group, the pyroptosis reached the peak at 24 h after modeling with the contents of active-cysteine-aspartic proteases-1 (caspase-1), Gasdermin D (GSDMD)-N, and cleaved-interleukin-1 beta (IL-1β) to the highest expression level. Meanwhile, we found that OMT (80 mg kg^−1^) remarkably decreased the expression levels of NLRP3, active-caspase-1, and cleaved-IL-1β at 24 h in the lesion tissue from UC rats. Further experiments on cells demonstrated that OMT at concentrations of 100 and 250 μM significantly inhibited cell death caused by NLRP3 inflammasome activation (*p* < 0.05), downregulated caspase-1, GSDMD, and decreased the levels of active-caspase-1, GSDMD-N, cleaved-IL-1β in RAW326.7 cells, and peritoneal macrophages. In summary, these results indicated that OMT could attenuate ulcerative colitis through inhibiting pyroptosis mediated by the NLRP3 inflammasome. The inhibition of the NLRP3 inflammasome may be a potential strategy for UC.

## 1. Introduction

Representing a chronic nonspecific inflammatory bowel disease with relapsing and remitting characteristics [1,2], UC is typically associated with symptoms such as abdominal pain, increased defecation frequency, muciform, and purulent or bloody diarrhea in the patients, which seriously affects the quality of patients’ lives. The recurrence of UC happens in up to 90% of patients, and the risk of cancer increases significantly with a long duration of UC [3]. However, the etiology of UC has not yet been fully understood, and the therapeutic strategies and drug development remain to be explored further.

Pyroptosis is a lytic programmed cell death initiated by the inflammasome, which responds to nonspecific immunity components, such as pathogen-associated molecular patterns (PAMPs) and damage-associated molecular patterns (DAMPs) [4]. This process involves plasma membrane rupture and pro-inflammatory cytokine release. The NLRP3 inflammasome senses various PAMPs or DAMPs generated by damaging agents such as toxins, pathogens, uric acid crystals, Adenosine Triphosphate (ATP), and so on, leading to its widespread involvement in the inflammatory response [5].

In active UC, NLRP3 is mainly expressed by neutrophils and other immune cells in the colonic lamina propria [6]. With proper stimulations, NLRP3 can join with Apoptosis-associated speck-like protein containing a CARD (ASC) and caspase-1 to form NLRP3 inflammasome, which then converts caspase-1 into an active form that can cleave GSDMD into GSDMD-N, a key executor of pyroptosis. This initiates pyroptosis by generating GSDMD-N and damaging the integrity of the cell membrane, leading to the release of cleaved IL-1β or interleukin-18 (IL-18) and causing inflammatory cell death [7]. The maturation and release of colonic IL-1β secreted by lamina propria macrophages depends on the inflammasome signaling pathways [8], and such IL-1β in UC patients accelerate disease activity [9,10]. Gene-deficient caspase-1 and NLRP3 have been shown to significantly alleviate symptoms of UC in mice [11,12], indicating less secretion of matured IL-1β. Although NLRP3-mediated pyroptosis has been identified as an important pathogenesis of UC, there are currently no therapeutic drugs targeting pyroptosis.

As one of the main components of *Sophora flavescens* Aiton in the Sophora genus of the Leguminosae family, OMT in experimental models show various effects such as analgesia, anti-inflammation, anti-oxidative stress, immunosuppression, anti-tumor, and so on. Meanwhile, OMT has been reported to improve the symptoms of the UC animal model [13,14,15,16,17]. However, whether OMT can regulate the NLRP3 inflammasome and pyroptosis still needs further investigation. Therefore, this study aimed to investigate the protective effect of OMT on UC and to reveal its targeting of pyroptosis mediated by the NLRP3 inflammasome. Both in vivo and in vitro experiments in this study have demonstrated that OMT has a significant inhibitory effect on pyroptosis mediated by the NLRP3 inflammasome. Therefore, OMT may be a potential therapeutic agent for UC by suppressing pyroptosis.

## 2. Results

### 2.1. Improvement in OMT on UC in Rats

**Figure 1:**
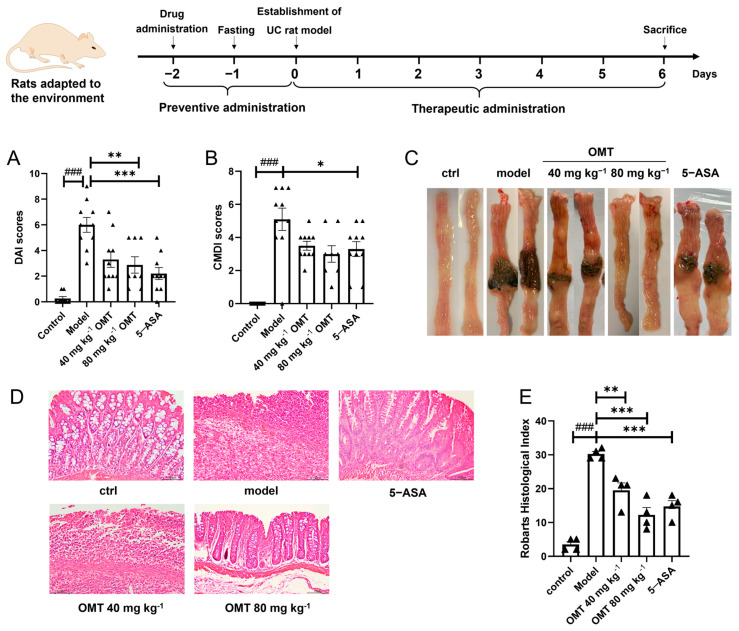
OMT alleviates symptoms and histopathological damage in TNBS-induced UC. (**A**) The DAI score was calculated by combining fecal properties, body weight changes, and fecal occult blood in rats. Model, TNBS-induced UC; OMT, oxymatrine treatment; 5-ASA, 5-aminosalicylic acid treatment. (**B**) The CMDI, which integrated the adhesion status and the severity of mucosal lesions, was used to assess the symptoms of ulcerative colitis in rats. (**C**) Representative anatomical images of colon in each treatment group on the sixth day of TNBS-induced UC. (**D**) Representative H&E staining of colonic lesions (scale bar: 100 μm). (**E**) RHI score was calculated according to the H&E staining results. Data are shown as mean ± SE. Compared to the model group, * *p* < 0.05, ** *p* < 0.01, *** *p* < 0.001; Compared to the control group, ### *p* < 0.001.

The experimental UC induced by TNBS in rats displayed characteristics similar to clinical symptoms, such as abdominal pain, diarrhea, bloody stools, ulcer edema, and granulocyte infiltration. In the present study, through observing the TNBS-induced UC rats, the DAI score was graded by integrating the changes in fecal morphology, occult blood, and body weight of rats, and the CMDI score was graded by recording the adhesion situation and ulcer severity at the time point of anatomy. The DAI and CMDI parameters were used to evaluate the improving effects of OMT on UC (Figure 1A,B). As expected, after colonal injection of TNBS, the rats developed severe abdominal pain, diarrhea, bloody stools, and severe ulcers in the colon. Compared to the control group, the DAI score (6.0 ± 0.6 vs. 0.3 ± 0.2, *p* < 0.01) and the CMDI score (5.1 ± 0.7 vs. 0, *p* < 0.01) of the model group increased remarkably. The ulcer area and symptoms in the 80 mg kg^−1^ OMT group showed significant improvement (DAI score: 2.9 ± 0.6, *p* < 0.01, CMDI score: 3.0 ± 0.5, *p* < 0.05), which was similar to the positive control drug 5-ASA (DAI score: 2.2 ± 0.5, *p* < 0.01, CMDI score: 3.3 ± 0.4, *p* < 0.05) (Figure 1A–C).

On the other hand, both OMT and 5-ASA improved the symptoms and colon pathological changes in rats (Figure 1D). The histopathological features of rats in the UC model group showed goblet cell defect in colonic epithelial tissue, a large number of inflammatory cells infiltration in the mucosa and submucosa, and granulation tissue hyperplasia. In contrast, 80 mg kg^−1^ OMT significantly improved TNBS-induced tissue damage, as demonstrated by a lower degree of columnar epithelial cell damage and lower granuloma and inflammatory infiltration. In addition, 40 mg kg^−1^ OMT also showed a certain improvement. According to the Robarts’ Histopathology Index scoring method, the pathological results were evaluated (Figure 1E). The RHI score of the UC model group was much higher than that of the control group (30.2 ± 0.8 vs. 3.5 ± 0.9) and was significantly reduced with 80 mg kg^−1^ OMT or 5-ASA treatment (12.2 ± 2.2 or 14.7 ± 1.7). This tendency was similar to the DAI and CMDI evaluation in relevant groups or treatments. This implies that OMT has therapeutic effects on ulcerative colitis, which is consistent with the previously reported results [4]. 

### 2.2. Development of NLRP3 Inflammasome-Mediated Pyroptosis and OMT Effects at the Early Stage of UC

**Figure 2:**
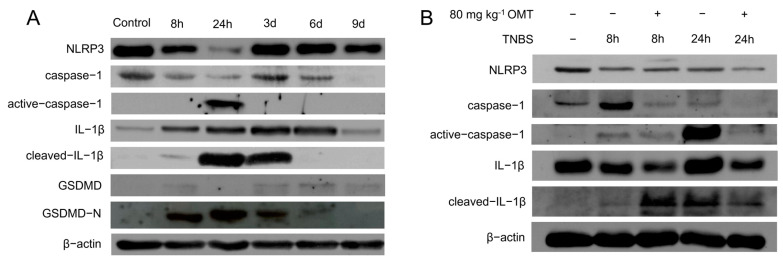
Changes in NLRP3 signaling pathway proteins in rats at different time points and the effects of OMT on early stage of UC after TNBS injection. (**A**) The protein expression levels of NLRP3 signaling pathway were detected at different time points after TNBS injection with the lesion tissues from UC rats. (**B**) The effects of 80 mg kg^−1^ OMT on the activation of NLRP3 signaling pathway at the early stage of UC were evaluated by measuring the expression levels of NLRP3, caspase-1, active-caspase-1, IL-1β, and cleaved-IL-1β at 8 h and 24 h after TNBS injection.

NLRP3 inflammasome activation is regulated by both “permit” and “activation” signals in vivo and may appear variously at different developing stages of UC. The activation of NLRP3 inflammasome is hallmarked by the production of active caspase-1, which can cleave GSDMD into GSDMD-N, the executor of pyroptosis. To obtain the activation pattern of NLRP3 inflammasome, the changes in protein expression on the NLRP3 inflammasome signaling pathway were detected from 8 h to 9 days after TNBS injection. In Figure 2A, the expression levels of NLRP3, caspase-1, IL-1β, and GSDMD varied at different injection time points from 8 h to 9 d after TNBS colonic administration, suggesting that the NLRP3 inflammasome might have played diverse roles across the stages of UC. The expression levels of GSDMD-N, active-caspase-1, and cleaved-IL-1β increased at 8 h and peaked at 24 h, indicating that the pyroptosis mediated by NLRP3 inflammasome activation mainly occurred within 24 h after TNBS injection. Moreover, the expression level of cleaved-IL-1β and GSDMD-N gradually decreased from 1 to 6 days, indicating the proceeding timeline of pyroptotic events.

Based on these results, we further examined the NLRP3 inflammasome activation-related protein levels at 8 h and 24 h after TNBS injection, respectively, aiming to reveal the effect of oxymatrine on the NLRP3 inflammasome-related pyroptosis at the early stage of UC. In Figure 2B, Western blot results showed that NLRP3, a sensor of stimuli, was significantly downregulated within 8 h after TNBS injection in UC rats, which might have reflected the protection of NLRP3 from overstimulation. Pre-administration of 80 mg kg^−1^ OMT inhibited the expressions of precursor protein caspase-1 and IL-1β after TNBS injection. The level of active-caspase-1, a key molecule in the NLRP3 inflammasome signaling pathway, significantly increased 24 h after TNBS injection but was significantly inhibited by OMT 80 mg kg^−1^ pre-administration. On the other hand, the cleaved IL-1β showed a different tendency with the OMT treatment at 8 h and 24 h after TNBS injection.

These results showed that OMT at an 80 mg kg^−1^ dosage significantly reduced the expression level of NLRP3 inflammasome signaling pathway-related proteins in the colon tissue of UC rats and suppressed the activation of caspase-1, thus preventing pyroptosis.

### 2.3. Effects of OMT on the NLRP3 Inflammasome Signaling Pathway and LPS/ATP-Orchestrated Pyroptosis in Macrophages

Given that the macrophages were involved in the changes in the microenvironment and development of UC, further experiments were carried out to explore the effects of OMT against pyroptosis on macrophages. As the priming signal stimulation and the activator of NLRP3 inflammasome, respectively, LPS and ATP co-operated the cellular events, including pyroptosis, following NLRP3 inflammasome activation. Therefore, the LPS/ATP stimulation was used to explore the OMT effects on the NLRP3 inflammasome-mediated pyroptosis in the macrophages.

**Figure 3:**
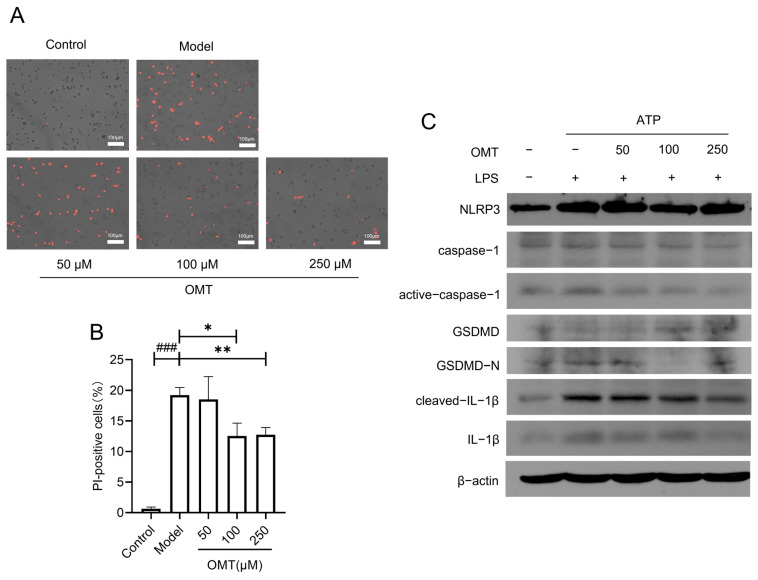
Effects of OMT on LPS/ATP-induced NLRP3-mediated pyroptosis in primary cultured rat peritoneal macrophages. (**A**) Representative images of PI staining of rat peritoneal macrophages (scale bar: 100 μm). Model, LPS/ATP stimulation with 3 mM ATP for 30 min after 10 μg mL^−1^ LPS pretreatment for 12 h; OMT, oxymatrine pretreatment for 1 h before LPS/ATP stimulation. (**B**) Percentage of PI-positive cells from primary peritoneal macrophage cultures (n = 4). Data are shown as mean ± SE. Compared to the model group, * *p* < 0.05, ** *p* < 0.01. Compared to the control group, ### *p* < 0.001. (**C**) Western blot analysis of the proteins of NLRP3 signaling pathway with the cultured rat peritoneal macrophage lysates.

In primary cultured rat peritoneal macrophages, LPS/ATP stimulation significantly induced more PI-positive staining, indicating cell pyroptosis with cell membrane damage. The treatment of OMT at 100 or 250 μM dramatically reduced the PI-positive cell ratio and inhibited the cell pyroptosis induced by LPS/ATP (*p* < 0.05) (Figure 3A,B). Western blotting method was performed to detect proteins related to NLRP3 inflammasome activation and pyroptosis, such as NLRP3, caspase-1, GSDMD, IL-1β, and their active forms in Figure 3C. LPS/ATP stimulation increased the expression levels of NLRP3 and IL-1β compared to the control in primary cultured peritoneal macrophages. Meanwhile, the protein levels of active caspase-1, as well as GSDMD-N and cleaved-IL-1β, were upregulated, which was reversed by the 100 and 250 μM OMT intervention (Figure 3C). These results revealed the anti-pyroptotic effect of OMT in the primary cultured peritoneal macrophages, which is consistent with that in PI staining.

**Figure 4:**
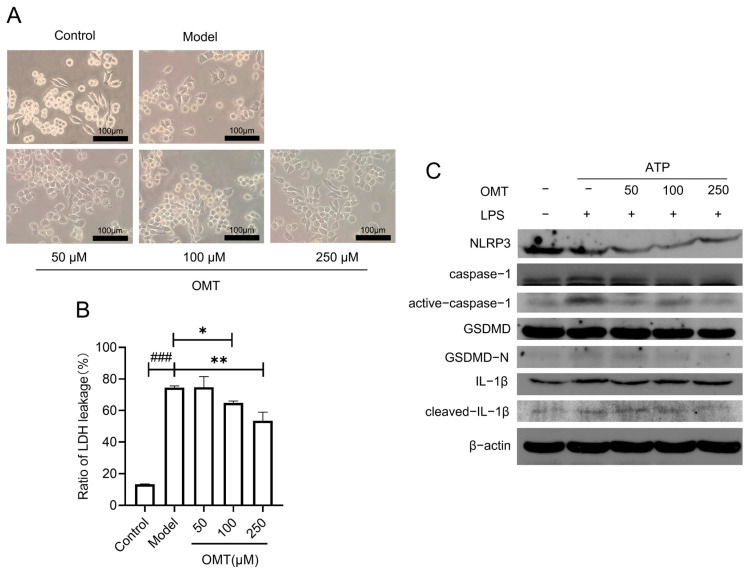
Effects of OMT on LPS/ATP-induced pyroptosis in RAW264.7 cells. (**A**) Representative images of the morphological changes in RAW264.7 cells in bright field under the LPS/ATP stimulation in the absence or presence of OMT (scale bar: 100 μm). Model, the LPS/ATP-stimulation. (**B**) Detection of the LDH release to determine the OMT effects on the LPS/ATP-induced cell damage. Data are shown as mean ± SE. Compared to the model group, * *p* < 0.05, ** *p* < 0.01. Compared to the control group, ### *p* < 0.001. (**C**) The changes in NLRP3 signaling-related protein levels in the RAW264.7 cell lysates under different conditions.

To further confirm the influence of OMT on the NLRP3 inflammasome activation and pyroptosis, experiments were carried out on the RAW264.7 macrophage cell line. After 30 min of ATP stimulation, babbles or protrusions were observed on the cell membrane in the LPS/ATP model group but not in the control group. This change was reversed by 100 μM and 250 μM but not 50 μM of OMT, and the cell morphology appeared rather normal even under the LPS/ATP stimulation (Figure 4A). Lactate dehydrogenase (LDH), a cytoplasmic enzyme, can be released out of a dying cell through the damaged membrane. In Figure 4B, LDH leakage was dramatically increased with LPS/ATP stimulation in RAW264.7 cells, and OMT pretreatment prevented this increase in a concentration-dependent manner, which is consistent with the result of PI staining in primary cultured peritoneal macrophages. As the hallmark of pyroptosis, protein levels of active-caspase-1, GSDMD-N, and cleaved-IL-1β were increased in the LPS/ATP model group compared with the control group in Figure 3C, suggesting that LPS/ATP-combined stimulation induced RAW264.7 cell pyroptosis. But, such increases were suppressed by OMT at a concentration of 100 or 250 μM. This indicated that 100 or 250 μM OMT treatment successfully inhibited LPS/ATP-orchestrated NLRP3-mediated pyroptosis of RAW264.7 cells.

## 3. Materials and Methods

### 3.1. Chemicals and Reagents

OMT (CAS: 16837-52-8, MF: C_15_H_24_N_2_O_2_, MW: 264.36) was purchased from Bidepharm (purity > 96%, Shanghai, China). A total of 5% TNBS aqueous solution was purchased from Sigma Chemical Co., Ltd. (St. Louis, MO, USA). The 5-aminosalicylic acid (5-ASA, CAS: 89-57-6, MF: C_7_H_7_NO_3_, MW: 153.14) and aprotinin were purchased from MACKLIN (Shanghai, China). DMEM high-glucose medium, DMEM/F-12 medium, Penicillin-Streptomycin liquid, Bicinchoninic acid (BCA) protein detection kit, Ethylenediaminetetraacetic acid (EDTA), N-2-hydroxyethylpiperazine-N-ethane-sulphonic acid (HEPES), Phosphate buffered saline (PBS), Lipopolysaccharides (LPS, Escherichia coli 055: B5), ATP, Radio immunoprecipitation assay (RIPA) lysis buffer, and ColorMixed protein marker were purchased from Solarbio (Shanghai, China). Phenylmethanesulfonyl fluoride (PMSF) was purchased from AMRESCO (Franklin Lakes, NJ, USA). Supersensitive enhanced chemiluminescence (ECL) reagent kits were purchased from Beyotime (Shanghai, China). Phosphate buffered saline (PBS) was purchased from Beijing Dingguo Changsheng Biotechnology Co., Ltd. (Beijing, China). Rabbit anti-caspase-1 polyclonal antibody, Rabbit anti-GSDMD polyclonal antibody, Rabbit anti-ASC polyclonal antibody, Rabbit anti-IL-1β polyclonal antibody, and HRP Goat Anti-Rabbit IgG and HRP Goat Anti-Mouse IgG were purchased from Abclonal (Wuhan, China). Rabbit anti-NLRP3 polyclonal antibody was purchased from Wanleibio (Shenyang, China). Mouse anti-β-actin monoclonal antibody was purchased from Bioworld (Dublin, OH, USA).

### 3.2. Animal Experiment

Specific Pathogen Free (SPF) Male Sprague Dawley (SD) rats weighing 240–250 g were purchased from Liaoning Changsheng Biotechnology Co., Ltd (Liaoning, China). (license approval number: SCXK (Liao) 2020-0001). The rats were well raised under controlled conditions with a temperature of 22 ± 2 °C, a 12-h light and dark cycle, and free access to food and water. They were then randomly assigned to groups of 7–10 individuals each for behavior study and sectioning experiments. All the animal experiments were carried out in strict accordance with the experimental procedures, and all animal experiments were approved by the Biomedical Ethics Committee of Dalian University of Technology (Approval No.: DUTSCE230728-02), Dalian City, Liaoning Province, China.

### 3.3. Establishment of UC Rat Model and Drug Administration

Before modeling, the rats were fasted for 24 h. After being anesthetized with an intraperitoneal injection of 10% chloral hydrate, the UC rat models were established by site-specific colonic injection of 35 mg kg^−1^ TNBS in 40% ethanol 8 cm away from the anus through a silicone tube. The rats’ behaviors were observed, and it was found that they normally developed obvious bloody stool and abdominal pain symptoms on the next day after modeling. For the treatment group, OMT was administrated via the anus daily, two days before TNBS injection. The research reported that oxymatrine was passively absorbed in the intestinal tract, and colonic absorption increased in a concentration-dependent manner as the administered dosage increased [18]. A positive control of 5-ASA suspension at a dose of 0.4 g kg^−1^ was administrated to the model rats via anal administration. In parallel, both the control and model rats received the same dosage of saline as a negative control.

### 3.4. UC Severity Evaluation and Histopathological Analysis

The rats were monitored for weight loss, fecal consistency, and fecal occult blood to assess disease activity index (DAI) [19], and the DAI score was calculated according to the criteria in Supplemental Table S1. The intraperitoneal adhesions and the appearance of colonic mucosal lesions were evaluated by the colonic mucosa damage index (CMDI) [20], which was based on the criteria provided in Supplemental Table S2.

Then, the histological study was carried out. In brief, the lesions of colon tissue were fixed with 4% paraformaldehyde tissue fixative, embedded in paraffin, and sliced at 5 μm thickness. Tissue sections were stained with hematoxylin-eosin (H&E) staining. Histological scores were calculated according to the Robarts’ Histopathology Index (RHI) scoring method [21] with the criteria outlined in Supplemental Table S3. The higher the score graded, the more severe the symptoms exhibited by the animal.

### 3.5. Culture of Macrophages

Primary peritoneal macrophages were prepared from rats as described below [22]. Briefly, rats were killed by carbon dioxide, and 10 mL of pre-cooled DMEM F-12 medium was injected intraperitoneally. The abdomen was then massaged for 5 min. The abdominal skin and peritoneum were separated layer by layer, and the peritoneal lavage fluid was collected. The collected lavage fluid was centrifuged for 10 min (4 °C, 1000 rpm), and cells in the pellet were suspended in a culture medium consisting of 10% FBS and 1% penicillin-streptomycin in DMEM F-12 medium. The cells were seeded at a density of 3.5 × 10^5^ cells/cm^2^ in culture dishes and cultured at 37 °C with 5% CO_2_ overnight. After being washed twice with PBS, the adherent cells were identified as peritoneal macrophages for further experiments.

The mouse macrophage RAW264.7 cells from China Center for Type Culture Collection (CCTCC, Shanghai, China) were cultured in the medium containing 10% FBS, 1% penicillin-streptomycin in DMEM high-glucose medium in the incubator at 37 °C with 5% CO_2_.

### 3.6. LPS/ATP-Orchestrated Pyroptosis and OMT Treatment in Macrophages

The LPS/ATP stimulation on macrophages was widely used in research on pyroptosis [23]. To investigate the effect of OMT pretreatment on LPS-induced pyroptosis, cells were pretreated with different concentrations of OMT (50, 100, or 250 μM) for 1 h. Then, the cells were exposed to LPS at a working concentration of 10 μg mL^−1^ for 12 h. After removing the medium containing LPS, the cells were further exposed to 3 mM ATP for 30 min in the serum-free culture medium.

For the propidium iodide (PI) staining experiment [24], primary peritoneal macrophages were exposed to 20 μg mL^−1^ PI in the presence of ATP. For the LDH assay [25], the medium of the RAW264.7 cell culture was collected to detect the released LDH using an LDH Activity Assay Kit, and absorbance at 490 nm was measured. The LPS/ATP-treated cells were used or harvested for further study. Each protocol was repeated at least three times.

### 3.7. Protein Extraction and Western Blot Analysis

Protein samples were prepared from colon tissues or cultured cells [26]. The colon tissue was homogenized in the homogenization buffer (EDTA 2 mM, HEPES 20 mM, pH = 7.5 containing aprotinin 1 μg mL^−1^, PMSF 1 mM), then centrifuged at 1700 rpm at 4 °C for 10 min. The cultured cells were lysed for 30 min on ice with RIPA lysis buffer containing 1 mM PMSF and 1 μg mL^−1^ aprotinin, then centrifuged at 10,000 rpm at 4 °C for 10 min. The supernatants were taken and stored at −20 °C for further experiments.

Protein concentration was detected with a BCA protein detection kit according to the manufacturer’s procedure, and a Western blot was performed to analyze targeting protein expressions. Briefly, the same amount of proteins in the extractions were SDS-sampled and separated on SDS-PAGE. Then, they were transferred to PVDF membranes. After being blocked at room temperature for 1 h in 5% semi-skimmed milk, the membrane was incubated with appropriate primary antibodies against NLRP3 (1:1500), caspase-1 (1:1000), GSDMD (1:1000), IL-1β (1:1000), or β-actin (1:10,000) overnight at 4 °C. Membrane was then rinsed with TBST and incubated with HRP-conjugated secondary antibodies (1:5000) for 1 h at room temperature. Finally, the blotted membrane was visualized with ECL reagent and detected with X-ray film.

### 3.8. Statistical Analysis

All statistical analyses were performed using GraphPad Prism 8.0. Data were presented as mean  ±  SE. The differences were assessed by a non-paired Student’s *t*-test between two groups and ANOVA analysis among three or more groups. *p*  <  0.05 was considered statistically significant.

## 4. Discussion

The inflammatory response triggered by pyroptosis of colonic lamina propria macrophages may be involved in the pathogenesis of UC. The release of cleaved-IL-1β during pyroptosis can upregulate NLRP3 expression in adjacent cells through the IL-1β-NF-κB-NLRP3 positive feedback [15]. The activation of NLRP3 leads to pyroptosis with the release of large amounts of cleaved-IL-1β and cleaved-IL-18, which can further cause serious inflammatory responses. Studies have shown that both NLRP3 and IL-1β are upregulated in colon biopsy samples of UC patients and are mainly localized in lamina propria cells dominated by neutrophils [6]. These findings imply that ulcerative colitis is related to the phenomena of NLRP3 inflammasome activation and subsequent pyroptosis. In the present study, we found that OMT improved the TNBS-induced ulcerative colitis in rats and alleviated the colonic mucosal lesions accompanied by the elevated Caspase-1, GSDMD, IL-1β, and reductions in NLRP3, caspase-1, and GSDMD. Therefore, we speculated that the therapeutic effects of OMT on UC are related to its inhibition of the NLRP3 inflammasome signaling pathway.

The NLRP3 inflammasome signaling pathway may play a key role in the occurrence and development of UC. The symptoms of ulcerative colitis were remarkably improved in NLRP3−/− mice treated with DSS or TNBS [27] or in wild-type mice treated with caspase-1 inhibitor after oral administration of DSS [12]. Correspondingly, spontaneous colitis occurred in IL-10−/− mice due to the caspase-1 and IL-1β release [28]. These findings suggested that NLRP3 signaling in UC was related to caspase-1 activation and subsequent secretion of inflammatory factor IL-1β. In this study, through analyzing the colon samples at different time points after TNBS intervention, we found that active-caspase-1, GSDMD-N, and cleaved-IL-1β increased simultaneously at the early stage of UC 24 h after modeling, suggesting that in addition to the NLRP3 inflammasome activation, pyroptosis might have also occurred during the onset of UC. Further investigation showed that OMT significantly reduced the levels of precursor proteins caspase-1, IL-1β, and mature proteins, active-caspase-1 and cleaved-IL-1β, in colon tissue 24 h after TNBS modeling. This suggests that OMT can inhibit UC by reducing the level of proteins required for NLRP3 inflammasome activation and initialing pyroptosis.

The NLRP3 inflammasome signaling pathway is under the control of dual signals, the “permit” and “activation” signals. The NF-κB activator is necessary for permission but not sufficient for NLRP3 activation. Meanwhile, the formation of pyroptotic spots accumulated by ASC protein cannot be observed under the stimulation with only NLRP3 agonist ATP [29]. Therefore, both the permissive signaling NF-κB and activation stimuli, such as ATP, are required to trigger pyroptosis.

Studies have shown that OMT could inhibit LPS-induced macrophage inflammation through the TLR4-NF-κB pathway [30]. OMT also has shown an immunosuppressive effect through suppressing NF-κB signaling in various diseases such as osteoarthritis [31] and neuritis [32]. This suggests that OMT can prevent the NF-κB permissive signaling of the NLRP3 inflammasome activation, but whether it can inhibit activation signaling of the NLRP3 inflammasome signaling pathway requires more in-depth studies.

**Figure 5:**
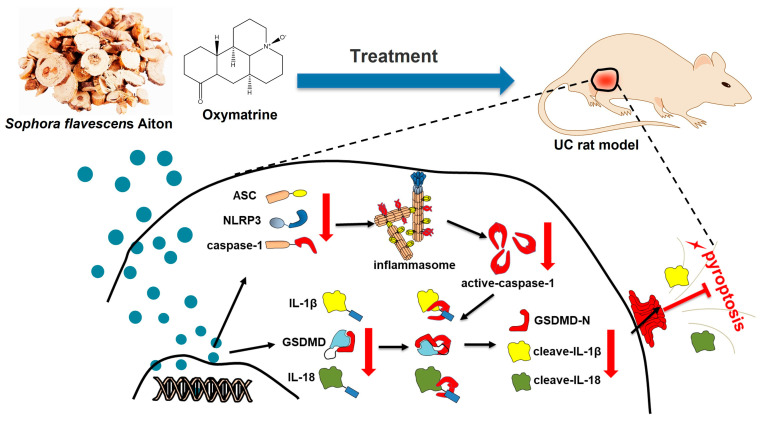
OMT attenuates UC through inhibiting pyroptosis mediated by the NLRP3 inflammasome. The blue arrow represents the drug treatment, the black arrow represents the protein formation process, and the red arrow represents the protein content decline.

To further analyze the inhibition of OMT on NLRP3 inflammasome signaling-mediated pyroptosis, an LPS/ATP induced-pyroptotic model on macrophage was used. LPS activates the TLR4-MyD88-NFκB signaling pathway as a “permission” signal [33], and ATP acts as an agonist of NLRP3 to provide an “activation” signal through activating the P2X7 receptor [34], which consequently upregulates the NLRP3 inflammasome signaling. Downstream of NLRP3 inflammasome activation, active-caspase-1 amplifies inflammation, which can cleave GSDMD to GSDMD-N, mediating cell death and cleave IL-1β, triggering wide inflammatory responses [35]. The present study showed that OMT inhibited cell death caused by LPS/ATP stimulation in the RAW264.7 cells and downregulated caspase-1 and GSDMD protein levels. We also found that the mature proteins, active-caspase-1, GSDMD-N, and cleaved IL-1β, were increased under the LPS/ATP stimulation in macrophage cells and were reversed by OMT treatment concentration-dependently. This further confirmed that OMT could prevent pyroptotic cell death mediated by the NLRP3 inflammasome signaling pathway. To summarize, results in this study suggest that OMT can regulate the expression of NLRP3, caspase-1, and GSDMD, inhibit the production of mature proteins, active-caspase-1, GSDMD-N, and cleaved-IL-1β, thus suppressing pyroptosis, thereby improving the symptoms of UC (Figure 5).

## 5. Conclusions

To the present, the decoction of Sophora flavescens Aiton has been proven to be therapeutic to UC patients, but the effects of its main alkali, OMT, are still under exploration. In this study, both in vivo and in vitro preclinical experiments have demonstrated that OMT had a significant inhibitory effect on pyroptosis mediated by the NLRP3 inflammasome, which suggests that OMT may be a potential therapeutic agent for UC by suppressing pyroptosis. The deposition kinetics of OMT under pathological conditions need to be further explored in the future.

## Data Availability

Data are contained within the article and Supplementary Materials.

## Supplementary Materials

The following supporting information can be downloaded at https://www.mdpi.com/article/10.3390/molecules29122897/s1. Supplementary Table S1: Disease Activity Index (DAI) scoring method; Supplementary Table S2: Colon mucosal injury index (CMDI) scoring method; Supplementary Table S3: Robarts Histopathology Score (RHI).
