# Supplementary material for: Oxymatrine Attenuates Ulcerative Colitis through Inhibiting Pyroptosis Mediated by the NLRP3 Inflammasome
Source: Molecules. 2024 Jun 18;29(12):2897. doi: 10.3390/molecules29122897 (PMC11206389; doi:10.3390/molecules29122897)
Supplement: Supplementary file 1 [file molecules-29-02897-s001.zip › molecules-3028564-supplementary.pdf]

Supplementary Material

1 Supplementary Table S1

Supplementary Table S1. Disease Activity Index (DAI) scoring method

Symptom	Evaluation Criteria	Score
Weight loss	No decrease;	0
	Decrease < 5%;	1
	Decrease from 5% to 10%;	2
	Decrease from 10% to 15%;	3
	Decrease of ≥15%;	4
Fecal property	Normal feces;	0
	Slightly poorly formed feces;	1
	Poorly formed feces;	2
	Loose feces;	3
	None feces	4
Fecal occult blood	Negative;	0
	Occult blood;	1
	Slightly fecal blood;	2
	Fecal blood	3

2 Supplementary Table S2

Supplementary Table S2. Colon mucosal injury index (CMDI) scoring method

Colon changes	Score
**ulceration and inflammation**	
No damage	0
Mild congestion, edema, smooth surface, no erosion or ulcer	1
Hemorrhagic edema, coarse granular mucosa, erosion or intestinal adhesion	2
Highly congestion and edema, mucosal surface necrosis and ulcer formation, ulcer area < 1 cm^2	3
Mucosal surface necrosis and ulcer formation or total intestinal wall necrosis, ulcer area is calculated for every 1cm^2 plus 1 score	4-8
**adhesion**	
None adhesion	0
Mild adhesion	1
Heavy adhesion	2

3 Supplementary Table S3

Supplementary Table S3. Robarts Histopathology Score (RHI)

Variable	Evaluation Criteria	Robarts Criteria
Chronic inflammatory infiltrate × 1	No increase	0
	Mild but unequivocal increase	1
	Moderate increase	2
	Marked increase	3
Lamina propria neutrophils × 2	No increase	0
	Mild but unequivocal increase	1
	Moderate increase	2
	Marked increase	3
Neutrophils in epithelium × 3	None	0
	≤5% crypts involved	1
	5% to 50% crypts involved	2
	≥50% crypts involved	3
Erosion or ulceration × 5	No erosion, ulceration or granulation tissue	0
	Recovering epithelium + adjacent inflammation	1
	Probable erosion focally stripped	1
	Unequivocal erosion	2
	Ulcer or granulation tissue	3
